# Effects of Maternal Obesity on Oxidative Parameters in Maternal and Cord Blood Samples

**DOI:** 10.7759/cureus.71303

**Published:** 2024-10-12

**Authors:** Gamze Gok, Ceylan Bal, Raziye Desdicioglu, Ayse Filiz Yavuz, Gulsen Yilmaz, Özcan Erel

**Affiliations:** 1 Medical Biochemistry, Ankara Bilkent City Hospital, Ankara, TUR; 2 Biochemistry, Ankara Yıldırım Beyazıt University Faculty of Medicine, Ankara, TUR; 3 Obstetrics and Gynecology, Ankara Yıldırım Beyazıt University Faculty of Medicine, Ankara, TUR

**Keywords:** cordal blood, maternal blood, obesity, oxidative stress, pregnancy

## Abstract

Aim

The purpose of this study was to analyze oxidative stress parameters in maternal and cord blood samples from both obese and nonobese women.

Methods

Our study included 30 obese and 35 nonobese pregnant women aged 18-40. We analyzed and compared oxidative stress parameters, including thiol/disulfide balance markers (native thiol, total thiol, and disulfide), albumin, ischemia-modified albumin (IMA), myeloperoxidase (MPO), catalase, ceruloplasmin, and intracellular glutathione levels.

Results

The comparison of maternal blood oxidative stress parameters between obese and nonobese pregnant women showed significantly higher levels of disulfide, catalase (kU/L), and ceruloplasmin (U/L) in the obese group (p = 0.005, p = 0.001, p < 0.001, respectively). Similarly, in cord blood, disulfide (µmol/L), IMA, catalase (kU/L), and (U/L) levels were significantly higher in the obese group (p < 0.001, p = 0.049, p < 0.001, p = 0.023, respectively), while albumin levels were significantly higher in the nonobese group (p = 0.003).

Conclusions

Our results suggest a strong association between maternal obesity and increased oxidative stress in both mothers and their offspring. Elevated oxidative stress levels may contribute to adverse maternal and fetal clinical outcomes. Therefore, we conclude that maintaining healthy weight control during reproductive age is crucial for ensuring maternal and fetal well-being.

## Introduction

Free radicals, which contain one or more unpaired electrons, are inherently unstable molecules. To achieve stability, they readily react with nearby molecules by accepting electrons from other atoms. While this process stabilizes the free radicals, it can cause damage to critical biological structures, such as DNA, proteins, and lipids. In healthy organisms, there is a balance between the production and elimination of free radicals, preventing cellular damage. However, when this balance shifts toward the excessive formation of free radicals, oxidative stress ensues, leading to potential harm [1,2].

Pregnancy is associated with increased sensitivity to oxidative stress. The amplified oxidative stress during pregnancy can result in various adverse outcomes for both the mother and fetus, including preeclampsia, recurrent pregnancy loss, placental dysfunction, fetal developmental anomalies, intrauterine growth restriction, and, in severe cases, fetal death [3].

WHO defines obesity as abnormal or excessive fat accumulation that poses a health risk [4]. BMI, calculated by dividing weight (kg) by height (m²), is a widely used measure to categorize obesity, with a BMI of 30 or higher indicating obesity. In recent decades, obesity has become a major public health concern, with the prevalence of excess body weight in adult women increasing from 24% to 40% between 1975 and 2016 [5,6].

Maternal obesity is well-established as a risk factor for both maternal and fetal morbidity [7]. It is associated with increased risks of gestational diabetes, preeclampsia, venous thromboembolism, cesarean section, postoperative wound infections, postpartum hemorrhage, and maternal mortality. Furthermore, it raises the likelihood of fetal complications, including macrosomia, preterm delivery, neonatal hypoglycemia, hyperbilirubinemia, congenital anomalies (such as neural tube defects, orofacial abnormalities, cardiac septal anomalies, and spina bifida), stillbirth, and perinatal mortality [8]. Maternal obesity has also been linked to elevated levels of inflammatory markers such as tumor necrosis factor-alpha, IL-1β, and IL-6 [9-11], with obese pregnant women exhibiting higher serum inflammatory marker levels than their nonobese counterparts [12]. Moreover, placental inflammatory markers and macrophage counts are more elevated in obese pregnant women [13], and these inflammatory markers promote oxidative stress through lipid peroxidation [14]. Some studies suggest that increased oxidative stress may adversely affect offspring [12,15,16].

Various biomarkers are used to assess oxidative stress, including reactive oxygen species, glutathione and its related enzymes (e.g., glutathione transferase, glutathione peroxidase, and glutathione reductase), malondialdehyde, oxidized glutathione, catalase, superoxide dismutase, oxidative stress index, and paraoxonase. Different pregnancy-related conditions have been associated with varying levels of oxidative stress markers [17].

In the present study, we hypothesized that obesity during pregnancy could induce oxidative stress in both the mother and the fetus. Our aim was to investigate the impact of maternal obesity on oxidative stress parameters in maternal and cordal blood, focusing on specific markers that have not been fully analyzed in this context, thus contributing to the existing literature.

## Materials and methods

Participants for the study were selected from those attending the Ankara Yıldırım Beyazıt University Atatürk Training and Research Hospital Obstetrics Outpatient Clinic. Approval was granted by the university’s ethics committee (22.07.2020/56). The target population included pregnant women aged 18-40 who conceived through regular intercourse. Exclusion criteria comprised multiple pregnancies, comorbidities, active smoking, and fetal distress. After obtaining informed consent, participants were divided into two groups based on their BMI: “obese” (BMI ≥30 kg/m²) and “nonobese” (BMI <30 kg/m²).

Blood samples from the mothers were taken during admission for delivery, and cord blood samples were collected from the umbilical cord immediately after birth. All samples were placed in whole blood collection tubes and serum separator tubes. The serum separator tubes were centrifuged at 1500 g for 10 minutes, and the supernatants were transferred to Eppendorf tubes. Samples in whole blood tubes were frozen until analysis. All analyses were performed using an autoanalyzer (Siemens Advia 1800, Siemens Healthcare GmbH, Henkestr, Germany), with chemicals sourced from Sigma-Aldrich (Sigma-Aldrich Lab&Production Materials, Merck KGaA, Darmstadt, Germany).

Thiol-disulfide homeostasis was measured using Erel and Neşelioğlu’s automatic spectrophotometric method [18]. Native thiol levels were measured using a kit containing DTNB (5,5′-dithiobis 2-nitrobenzoic acid), and total thiol (the sum of native and reduced thiol) was measured using a second kit. The disulfide value was calculated as half the difference between total and native thiol. Albumin levels were determined using the bromocresol green assay, as described by Doumas et al. [19], with endpoint measurements taken at 596/694 nm.

Serum ischemia-modified albumin (IMA) levels were assessed using a rapid colorimetric method developed by Bar-Or et al. [20]. Specimen absorbance was measured at 470 nm, and results were expressed in absorbance units (ABSU). Myeloperoxidase (MPO) activity was measured using hydrogen peroxide and o-dianisidine, following Bradley et al.’s method [21], with absorbance readings at 460 nm. Serum ceruloplasmin ferroxidase activity was evaluated based on Erel’s method [22], while catalase enzyme activity was determined using Goth’s modified spectrophotometric technique [23].

Hemoglobin was measured using the cyanmethemoglobin method with Drabkin’s Reagent [24]. Glutathione levels were determined by Alisik et al.’s method [25]. Blood samples were collected in tubes containing ethylenediaminetetraacetic acid, and glutathione was measured both before and after reduction. Oxidized glutathione was calculated as half the difference between total and native glutathione, expressed as μmol/g hemoglobin.

Statistical analysis

All statistical analyses were performed using IBM SPSS Statistics for Windows, Version 22.0 (Released 2013; IBM Corp., Armonk, NY, USA) Data were expressed as means and SDs. The Shapiro-Wilk test was employed to assess the normality of the data distribution. For normally distributed data, the independent samples t-test was applied, while the Mann-Whitney U test was used for non-normally distributed variables. A p-value of less than 0.05 was considered statistically significant.

## Results

The study involved 30 obese and 35 nonobese pregnant participants. The mean age was 27.23 ± 4.17 years for obese participants and 28.20 ± 5.18 years for nonobese participants. The mean BMI values were 32.27 kg/m² for the obese group and 26.42 kg/m² for the nonobese group.

A comparison of oxidative stress parameters in maternal blood between the obese and nonobese participants revealed that disulfide, catalase (kU/L), and ceruloplasmin (U/L) levels were significantly higher in the obese group (p = 0.005, p = 0.001, p < 0.001, respectively) (Table 1).

**Table 1:** Results of the maternal blood oxidative stress parameter analysis of nonobese and obese pregnant women For normally distributed values, the independent samples t-test was employed, while the Mann-Whitney U test was utilized for parameters that did not follow a normal distribution. * A p-value of less than 0.05 was considered statistically significant. ABSU, absorbance units; IMA, ischemia-modified albumin; MPO, myeloperoxidase

Parameters	Maternal blood samples of nonobese pregnant women	Maternal blood samples of obese pregnant women	p-value
Native thiol µmol/L	319.7 (151.5-482)	313.5 (128.4-496.8)	0.35
Total thiol µmol/L	352.1 (166.9-530.8)	358.32 (146.7-590.9)	0.683
Disulfide µmol/L	16.68 ± 4.11	22.04 ± 8.86	0.005*
Albumin g/dL	3.92 ± 0.80	3.65 ± 0.98	0.23
IMA ABSU	0.66 ± 0.29	0.75 ± 0.32	0.276
Catalase kU/L	105.4 (49.48-139.9)	118.45 (20.79-218.5)	0.001*
MPO U/L	104.11 ± 22.94	109.39 ± 26.45	0.393
Ceruloplasmin U/L	512.5 (492.2-536.3)	553.2 (493.3-595.9)	<0.001*
Native glutathione as per gram hemoglobin (µmol/g Hb)	59.84 (31.64-186.9)	59.84 (23.27-206.5)	0.927
Total glutathione as per gram hemoglobin (µmol/g Hb)	76.46 (41.80-248.7)	75.21 (35.71-248.9)	0.854
Disulfide as per gram hemoglobin (µmol/g Hb)	8.35 (3.65-39.69)	8.05 (3.64-21.17)	0.486

The comparison of oxidative stress markers in cord blood samples between obese and nonobese pregnant women revealed that levels of disulfide (µmol/L), IMA (ABSU), catalase (kU/L), and MPO (U/L) were significantly higher in the obese group (p < 0.001, p = 0.049, p < 0.001, p = 0.023). Conversely, albumin levels (g/dL) were significantly elevated in the nonobese pregnant women (p = 0.003) (Table 2).

**Table 2:** Results of the cordal blood oxidative stress parameter analysis of nonobese and obese pregnant women For normally distributed values, the independent samples t-test was employed, while the Mann-Whitney U test was utilized for parameters that were not normally distributed. * A p-value of less than 0.05 was considered statistically significant. ABSU, absorbance units; IMA, ischemia-modified albumin; MPO, myeloperoxidase

Parameters	Cordal blood samples of nonobese pregnant women	Cordal blood samples of obese pregnant women	p-value
Native thiol µmol/L	353.4 (236.9-397.7)	335.4 (224.9-386.5)	0.053
Total thiol µmol/L	400 (295.8-438.3)	392.6 (277.7-445.4)	0.797
Disulfide µmol/L	20.68 ± 4.71	27.16 ± 6.31	<0.001*
Albumin g/dL	4.06 ± 0.42	3.77 ± 0.31	0.003*
IMA ABSU	0.734 ± 0.14	0.80 ± 0.12	0.049*
Catalase kU/L	116.4 (91.47-142.8)	126.8 (104-168.4)	<0.001*
MPO U/L	117.6 ± 19.71	129.8 ± 22.46	0.023*
Ceruloplasmin U/L	558.1 (387.2-718.7)	603.3 (438.5-760.3)	0.067
Native glutathione as per gram hemoglobin (µmol/g Hb)	42.15 (24.47-106.7)	39.80 (23.98-82.98)	0.622
Total glutathione as per gram hemoglobin (µmol/g Hb)	55.21 (28.94-128.1)	54.25 (31.87-96.91)	0.693
Disulfide as per gram hemoglobin (µmol/g Hb)	6.4 (2.23-21.38)	6.71 (2.09-12.64)	0.549

## Discussion

A healthy pregnancy is associated with oxidative stress due to placental activity and increased oxygen consumption [2]. This oxidative stress, “controlled” by antioxidative systems, is essential for maintaining pregnancy, contributing to placental angiogenesis and trophoblast proliferation [2,26]. However, obesity can also induce oxidative stress, potentially overwhelming these antioxidative mechanisms and leading to unfavorable outcomes [16]. It has been suggested that oxidative stress related to maternal obesity increases the risks of spontaneous abortion, miscarriage, preeclampsia, and congenital malformations [2]. Furthermore, obesity may lead to gestational diabetes mellitus and insulin resistance [17].

Oxidative stress arising from maternal obesity can cause direct fetal DNA damage and lead to congenital malformations by inhibiting the PAX3 gene, a transcription factor crucial for neural tube formation. Thiols, also known as mercaptans, are sulfhydryl-containing organic compounds involved in oxidation-reduction reactions. The balance of thiol-disulfide can be used to assess oxidative stress, as thiols form disulfide bonds in oxidized states. This dynamic balance plays a critical role in pregnancy. In a study conducted by Katar-Yıldırım et al., both native and total thiol levels were lower in pregnant women at risk for threatened abortion compared to those without such risk [27]. Aktun et al. compared cord blood parameters between diabetic and nondiabetic pregnant women, concluding that disulfide levels were elevated in the former group [28]. Another study found that both serum native and total thiol levels were lower in pregnant women with preeclampsia compared to those without [29]. Additionally, Eroğlu et al. reported that the thiol-disulfide profile could predict fetal development and assess the severity of fetal growth retardation [30]. Ozler et al. investigated the effects of obesity and gestational diabetes on thiol/disulfide homeostasis in cord blood, finding significantly higher disulfide levels in these individuals compared to controls [31]. Similarly, our findings revealed elevated cord blood disulfide levels in obese pregnant women compared to their nonobese counterparts, indicating that offspring of obese mothers are exposed to increased oxidative stress. These results align with existing literature on thiol/disulfide homeostasis.

In our study, we analyzed the impact of obesity on thiol/disulfide homeostasis in both maternal and cord blood. We found that disulfide levels were higher in the maternal blood of obese mothers compared to nonobese mothers, indicating increased oxidative stress in both the mothers and their offspring.

Albumin, an acute-phase reactant, reflects an individual’s nutritional status [32] and acts as an antioxidant scavenger of free radicals. The N-terminal region of the albumin molecule contains the aspartate-alanine-histidine-lysine amino acid sequence, which binds to nucleic acids and divalent heavy metals, including nickel, copper, and cobalt [33]. However, free radicals can damage the N-terminal of the albumin molecule, impairing its ability to bind these metals. This altered form of albumin, characterized by structural changes due to hydroxyl radicals in its N-terminal, is referred to as IMA [20,34]. Our comparison of cord blood parameters between nonobese and obese pregnant women revealed higher albumin levels in the former and elevated IMA levels in the latter. These findings suggest that maternal obesity may expose offspring to oxidative stress.

Glutathione is the most prevalent intracellular antioxidant, containing a thiol group. Under oxidative stress, the reduced form of glutathione can be oxidized. It plays a crucial role in determining intracellular redox potential [25]. Loukidi et al. measured reduced glutathione levels in the erythrocytes of obese and nonobese pregnant women, finding them to be lower in the former group [35]. Sen et al. analyzed oxidized and reduced glutathione levels in maternal blood samples of 15 obese and 15 nonobese pregnant women during the 24th and 28th weeks of pregnancy, reporting a higher oxidized to reduced glutathione ratio in obese pregnant women [36]. Malti et al. assessed maternal and cord blood samples from 40 obese and 50 nonobese pregnant women regarding reduced glutathione levels, revealing lower maternal blood levels in obese women, but no significant difference in cord blood samples [16]. In our study, we measured glutathione levels following the method described by Alisik et al. [25]. In line with Malti et al., our analysis did not reveal significant differences between obese and nonobese pregnant women concerning maternal blood glutathione levels [16].

Ceruloplasmin, a copper-containing molecule, constitutes the major part of circulating copper. It plays roles in coagulation, angiogenesis, iron metabolism, and the regulation of oxidative stress. Kim et al. reported that increased plasma ceruloplasmin levels are associated with obesity [37]. Cignarelli et al. found that ceruloplasmin levels were higher in obese participants than in those with normal weight [38]. Our comparison of cord blood ceruloplasmin levels between obese and nonobese mothers did not show significant differences; however, maternal blood ceruloplasmin levels were significantly higher in obese mothers. This finding indicates that obese pregnant women experience increased oxidative stress.

Catalase, one of the first discovered antioxidant enzymes, is involved in hydrogen peroxide metabolism and prevents its intracellular accumulation, thus protecting cells from oxidative damage [39]. A study investigating catalase activity in maternal and cord blood samples and placental tissues of obese and nonobese pregnant women found higher catalase activity in the cord blood and placental tissues of obese mothers [16]. Our study similarly found that catalase activity was higher in the maternal and cord blood samples of obese mothers compared to nonobese ones. We propose that the increased antioxidant activity detected in our analysis reflects an adaptive response to heightened oxidative stress associated with obesity.

MPO, expressed in neutrophils and monocytes, is a member of the peroxidase family involved in antimicrobial activities and the defense mechanisms of neutrophils. Elevated MPO levels in circulation are associated with inflammation and increased oxidative stress [40]. Zaki et al. noted that obesity correlates with increased MPO levels [41]. While our study found no significant difference in maternal blood MPO levels between obese and nonobese pregnant women, MPO levels in the cord blood of obese pregnant women were higher than those of nonobese women. We suggest that increased MPO levels in the cord blood samples of obese pregnant women may be an antioxidative response to heightened oxidative stress in their offspring.

Obesity is a chronic and multifactorial condition characterized by increased fat accumulation in the body. Triglycerides are stored in fat tissue, and substances known as adipokines are produced in white fat tissue. IL-6, an adipokine, plays a role in weight control through its inflammatory functions, influencing the leptin hormone that stimulates dopamine uptake and induces feelings of satiety. Furthermore, adipokines contribute to oxidative stress by increasing reactive oxygen species. The increase in fat tissue leads to diminished effectiveness of antioxidant systems and heightened oxidant system effects [42]. Our study’s results align with mechanisms supporting the increase in oxidative stress due to adipose tissue accumulation.

Our findings indicate that maternal obesity leads to increased oxidative stress in both mothers and their fetuses. To our knowledge, this is the first study to analyze thiol-disulfide, intracellular glutathione, albumin, IMA, ceruloplasmin, and MPO parameters in cord and maternal blood samples of obese and nonobese pregnant women. We believe this research significantly contributes to the literature.

## Conclusions

Over the past few decades, obesity has significantly increased among reproductive-aged women. Our study indicates that maternal obesity contributes to elevated oxidative stress in both mothers and their offspring. Given the association between increased oxidative stress and adverse maternal and fetal clinical outcomes, effective weight management is crucial for promoting the health of both mothers and their children during reproductive years.
